# Photocatalyzed CO_2_ reduction to CO by supramolecular photocatalysts made of Ru(II) photosensitizers and Re(I) catalytic subunits containing preformed CO_2_TEOA adducts

**DOI:** 10.1038/s41598-023-38411-3

**Published:** 2023-07-13

**Authors:** Antonio Santoro, Ambra M. Cancelliere, Kei Kamogawa, Scolastica Serroni, Fausto Puntoriero, Yusuke Tamaki, Sebastiano Campagna, Osamu Ishitani

**Affiliations:** 1 Department of Chemical, Biological, Pharmaceutical and Environmental Sciences, University of Messina, and Interuniversitary Research Center for Artificial Photosynthesis (Solar Chem, Messina Node), V. F. Stagno d’Alcontres 31, 98166 Messina, Italy; 2 Department of Chemistry, School of Science, Tokyo Institute of Technology, 2-12-1-NE-2 O-okayama, Meguro-Ku, Tokyo 152-8550 Japan; 3 Research Institute for Chemical Process Technology, Department of Materials and Chemistry, National Institute of Advanced Industrial Science and Technology (AIST), 4-2-1 Nigatake, Miyagino-Ku, Sendai, Miyagi 983-8551 Japan; 4 Department of Chemistry, Graduate School of Advanced Science and Engineering, Hiroshima University, 1-3-1 Kagamiyama, Higashi-Hiroshima, Hiroshima, 739 8526 Japan

**Keywords:** Photocatalysis, Photocatalysis

## Abstract

Two new supramolecular photocatalysts containing Ru(II) polypyridine units as light-harvesting photosensitizers and Re(I) polypyridine subunits as catalytic centers have been prepared. The new species, **RuRe2A** and **Ru2ReA**, contain catalytic Re(I) subunits coordinated by the preformed CO_2_TEOA adduct (known to be the effective catalytic subunits; TEOA is triethanolamine) and exhibit quite efficient and selective photoreduction of CO_2_ to CO, with outstanding TONs of 2368 and 2695 and a selectivity of 99.9% and 98.9%, respectively. Such photocatalytic properties are significantly improved with respect to those of previously studied **RuRe2** and **Ru2Re** parent compounds, containing chloride ligands instead of the CO_2_TEOA adduct. Comparison between photocatalytic performance of the new species and their parent compounds allows to investigate the effect of the CO_2_TEOA insertion process as well as the eventual effect of the presence of chloride ions in solution on the photocatalytic processes. The improved photocatalytic properties of **RuRe2A** and **Ru2ReA** compared with their parent species are attributed to a combined effect of different distribution of the one-electron reduced form of the supramolecular photocatalysts on the Ru-subunit(s) (leading to decreased CO formation due to a poisoning ligand loss process) and on the Re-subunit(s) and to the presence of chloride ions in solution for **RuRe2** and **Ru2Re**, which could interfere with the CO_2_TEOA adduct formation, a needed requisite for CO forming catalysis. These results strongly indicate the utility of preparing supramolecular photocatalysts containing preformed adducts.

## Introduction

The large use of fossil fuels as the main energy source has led to the release of more than 36 billions of tons of CO_2_ per year into the atmosphere^1^. The increasing amount of CO_2_ is the main driver for global important issues like earth warming and climate changes. As a result, it becomes essential for human beings to find new and sustainable ways to produce energy without further CO_2_ production. The environmentally clean, homogeneously distributed, abundant and inexpensive nature of sunlight makes it a promising candidate to be a renewable energy source.

In this general framework, a major research challenge consists in designing abiotic photocatalytic systems capable of producing energy-rich chemicals by photochemical reduction of CO_2_^2^. Conversion of CO_2_ into CO and HCOOH with the aid of solar energy input appears interesting from different points of view: (i) reduces global warming, by increasing the use of solar energy, (ii) transforms a pollutant as CO_2_ into an energy resource, (iii) sets up models for the study of natural photosynthesis^2–4^. The synergic interaction of a light-harvesting photosensitizer (PS)—whose role is absorption of solar energy—and a catalyst (CAT)—having the role of performing catalytic processes, when activated by PS—is quite useful to achieve an efficient photocatalytic reduction of CO_2_. For such reasons, it is instrumental to develop multicomponent, supramolecular photocatalysts made of photosensitizers and catalysts, suitably designed to achieve a fast and efficient electron transfer from the one electron reduced species of PS unit—photochemically excited and then reduced by using a sacrificial reducing agent—to the CAT^5^. In this context, Ru(II) polypyridine complexes are widely employed as PS in the field of solar energy conversion^6–14^, thanks to the combination of different properties: (i) strong absorption in the visible region (that is, an efficient light harvesting); (ii) relatively long lifetime of the excited state; (iii) strong oxidation power in the excited state; (iv) high stability of both ground state and one-electron reduced state^15–18^**.** At the same time, an efficient CAT should show high selectivity of CO_2_ reduction versus the competitive formation of H_2_, high quantum yield, high turnover number and turnover frequency. In this regard, many Re(I) diamine carbonyl complexes have been studied^5, 7, 19–26^.

Among all the possible approaches to achieve photo-assisted CO_2_ reduction, the one that provides the use of multinuclear complexes, i.e., supramolecular photocatalysts, incorporating Ru(II)-polypyridine subunits as the PS and Re(I) diamine complexes as the CAT units looks one of the most promising^5^. The advantages of supramolecular photocatalysts compared to a mixed system of the corresponding mononuclear metal complexes derive from the acceleration of the photoinduced electron transfer between its components, not limited by diffusion. Such behaviour leads to improved performance and higher durability of the photocatalytic system, mainly due to the fast scavenging of the photosensitizer reduced state. In the supramolecular photocatalysts, PS and CAT subunits are held together by a bridging ligand (BL), whose nature and length influence electronic coupling and as a consequence electron transfer rate constants, and therefore has a strong impact on the photocatalytic properties of the assembly^5, 24^.

**Chart 1:**
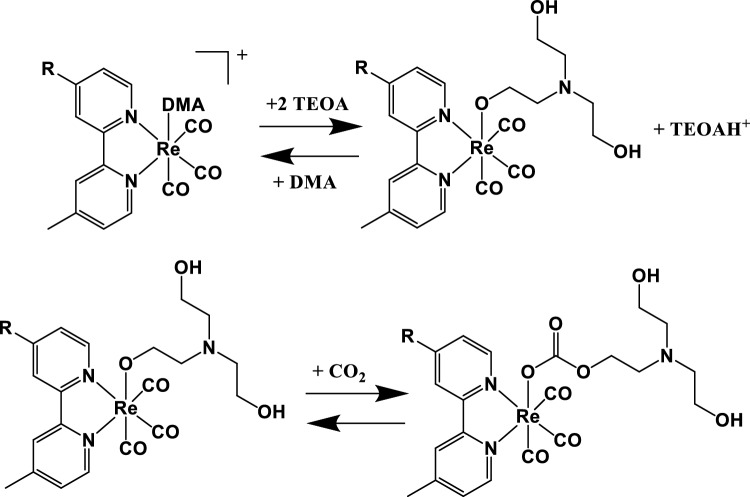
General scheme of CO_2_TEOA insertion in Re(LL)(CO)_3_X complexes (LL = polypyridine; X = halides or other substituents), after replacement of the X ligand with DMA^22, 23^. R represents a generic substituent, including eventual connections with other subunits like PS components.

Indeed, in the photocatalytic CO_2_ reduction process involving most of Re(I) polypyridine complexes as the catalytic species (or catalytic subunits, in the case of supramolecular photocatalysts), the active species is the Re-CO_2_TEOA adduct (TEOA = triethanolamine)^21–24^, so the initial step of the photocatalytic process in Re(I) photocatalytic complexes is ligand replacement and CO_2_-TEOA adduct formation (see Chart 1). This can also have a role in the overall photocatalytic process, for example if competitive reactions to CO_2_-TEOA insertion can take place, or however by delaying the photocatalytic activity. In literature, most reported data refer to experiments performed on Re(LL)(CO)_3_X (LL = polypyridine; X = halides or other substituents) subunits^7^, whereas other recent data are referred to compounds containing Re(LL)(CO)_3_(CO_2_TEOA)^+^ (LL = polypyridine ligand) catalytic species^27^. Even when both halides or CO_2_TEOA containing Re(I) complexes are reported in the same work^22, 24, 28^, direct comparison of their photocatalytic properties are not reported, so the eventual effect of the CO_2_TEOA insertion process on the photocatalysis has not been faced. This strongly limits any direct comparison among different results reported in literature.

**Figure 1:**
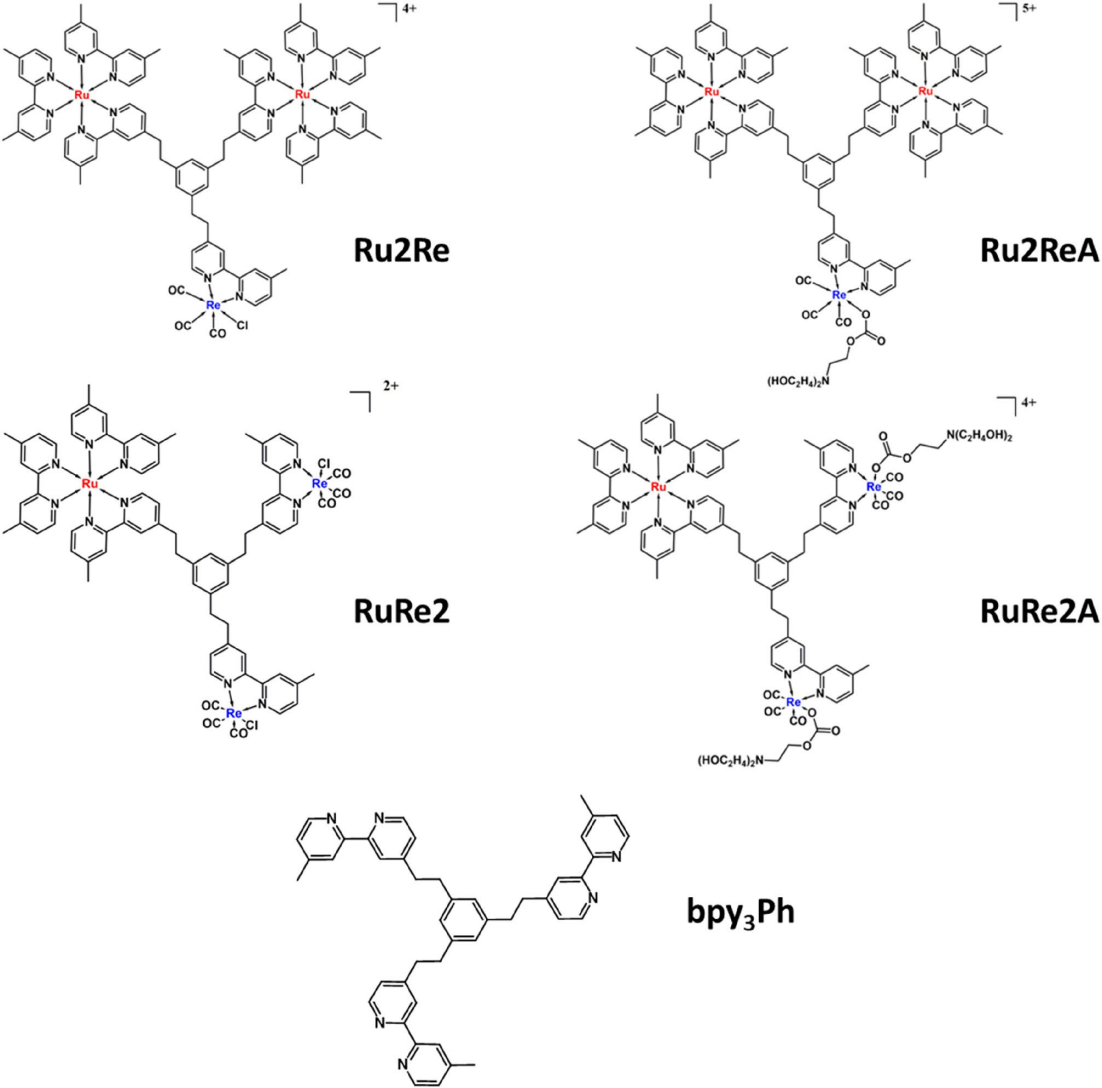
Structural formulas of the studied **Ru2ReA** and **RuRe2A** supramolecular photocatalysts. The parent reference compounds **Ru2Re** and **RuRe2** and the bridging ligand **bpy**_**2**_**Ph** are also shown. All the metal complexes have been prepared and studied as hexafluorophosphate salts.

We recently proposed a series of supramolecular photocatalysts, **Ru2Re** and **RuRe2,** where one (or two, respectively) PS subunits were connected to two (or one) CATs, by a new BL (**bpy**_**3**_**Ph**, see Fig. 1)^29^. The Ru(II) polypyridine species and the Re(I) subunits, employed as PS and CAT units respectively, maintained their light absorption and redox properties once connected through the BL, and their assemblies showed quite efficient light induced CO formation, high stability and selectivity, together with high turnover numbers for CO photoproduction (TON)^29^. The photocatalysts operated in CO_2_-saturated mixed solution of *N,N*-dimethylacetamide–triethanolamine (DMA–TEOA 5:1 v/v); therefore, the initially coordinated chlorides of the Re(I) subunit(s) were replaced by CO_2_TEOA insertion, however chloride ions remain present in solutions and the effect (if any) of the CO_2_TEOA insertion process on the rate of the photocatalysis was not revealed.

In the present paper, we studied an evolution of the two formerly investigated species **Ru2Re** and **RuRe2**, in which the chloride ligands of the Re(I) catalytic subunit(s) are replaced by –CO_2_TEOA groups, the chloride ions are removed from the solution and photocatalysis starts with preformed adducts (compounds **Ru2ReA** and **RuRe2A**, shown in Fig. 1). Direct comparison between the photocatalytic properties of the two new compounds **Ru2ReA** and **RuRe2A** with those of the formerly prepared **Ru2Re** and **RuRe2** species also allows to investigate the effect of eliminating the ligand replacement step, disentangling such an effect from other effects due to different compositions of the supramolecular Ru-Re species apart from substituting chloride with CO_2_TEOA in the coordination sphere of the Re(I)-based catalyst subunit. The results indicate the advantage of using preformed TEOA-CO_2_ adducts in Re(I) catalyst subunits for improving the photocatalytic properties of supramolecular photocatalysts as far as the reduction of CO_2_ is concerned.

## Results and discussion

### Synthesis

To prepare the new photocatalytically-active **Ru2ReA** and **RuRe2A** species, a well-established protocol procedure was applied^22, 23, 27^. Typically, **Ru2Re** or **RuRe2**^29^ were dissolved in acetonitrile-H_2_O (4:1 v/v) containing saturated NH_4_PF_6_, and the solutions were left under stirring for 8 days in the dark at room temperature. During this time window, the acetonitrile takes the place of the chloride anion in the coordination sphere of the Re(I) subunit(s). The acetonitrile coordinated complex so obtained was filtrated off, recrystallized from a CH_2_Cl_2_–Et_2_O mixed solution as hexafluorophosphate salt, and dried under vacuum. This procedure allows to eliminate the leaving chloride anions from the solution, and this is the only relevant modification to the useful protocol. The filtrated powder was then dissolved in 25 mL of *N,N*-dimethylacetamide (DMA) and left in the dark for other 5 h. In such a period the DMA adduct was formed by substitution of acetonitrile. Afterwards, 5 mL of triethanolamine (TEOA) were added and the solution was left in the dark overnight to finally obtain the TEOA adduct. The TEOA adducts are the precursor of the CO_2_TEOA complexes **Ru2ReA** and **RuRe2A**, which were obtained during the preparation of the sample for the photophysical experiments, by bubbling CO_2_ for 20 min in the solution containing the supramolecular photocatalysts.

### Photocatalytic experiments

#### General scheme of photocatalysis

The sequence of events occurring in the photocatalytic process that involves any supramolecular photocatalyst containing Ru(II)-based photosensitizers and Re(I)-based catalysts, generally named Ru-Re, is schematized in Eqs. (1)–(5)^5^. This behavior is assumed reasonably valid for the supramolecular photocatalysts here discussed. In such a process, the photoexcitation [Eq. (1)] and the subsequent reduction [Eq. (2)] of the ruthenium moiety by the sacrificial reagent 1,3-dimethyl-2-phenyl-2,3-dihydro-1H-benzo[d]imidazole (BIH, a bielectronic reducing species) leads to the Ru-based one-electron reduced species (OERS) of the photocatalyst and the formation of BI^**.**^, the radical form of BIH. Then, an intramolecular electron transfer occurs from the ruthenium to rhenium subunits giving the one-electron reduced form of the Re(I) catalytic active species [Eq. (3)], able to reduce CO_2_ to CO, once doubly-reduced [Eq. (4)]. Actually, such reduction process consists in a two-electrons process in which the second negative charge [“*e*^*−*^ ” in Eq. (4)] can be provided by another catalyst present in solution in its OERS, formed by the sequence of events shown in Eqs. (1)–(3) or produced by reduction of Ru-Re by the radical BI^**.**^ formed by deprotonation of BIH^·+^ [Eq. (5)], which shows a reducing power (E_p_^ox^ =  − 2.06 V vs Fc^+^/Fc) strong enough to donate an electron directly to the ground state of ruthenium moiety of the photocatalyst^5^. Actually, the OERS of Ru-Re can also be directly reduced by the radical BI^**.**^ species. Some HCO_3_^−^ is also formed, and its amount is almost equivalent to that of CO^5^, supporting the photocatalytic scheme.

1 $${\text{Ru}}\! -\!{\text{Re }} + {\text{ h}}\nu \to *{\text{Ru}}\! -\!{\text{Re}}$$

2 $$*{\text{Ru}}\! -\!{\text{Re }} + {\text{ BIH}} \to {\text{Ru}}^{ - }\! -\!{\text{Re}} + {\text{BI}}^{ \cdot } + {\text{H}}^{ + }$$

3 $${\text{Ru}}^{ - }\! -\!{\text{Re}} \to {\text{Ru}}\! -\!{\text{Re}}^{ - }$$

4 $${\text{Ru}}\! -\!{\text{Re}}^{ - } + e^{ - } + {\text{ H}}^{ + } + {\text{ 2 CO}}_{{2}} \to {\text{Ru}}\! -\!{\text{Re }} + {\text{ CO }} + {\text{ HCO}}_{{3}}^{ - }$$

5 $${\text{Ru}}\!-\!{\text{Re }} + {\text{ BI}}^{ \cdot } \to {\text{Ru}}^{ - }\!-\! {\text{Re }}({\text{or }}\;{\text{Ru}}\!-\!{\text{Re}}^{ - } ) + {\text{BI}}^{ + }$$

#### General procedure

In order to achieve a similar light-absorption features and compare the results obtained in the various experiments for **RuRe2A** and **Ru2ReA**, for the photocatalytic experiments the solutions were prepared by maintaining a roughly similar concentration of the Ru-subunit photosensitizer, i.e. [Ru_2_Re] ca. 25 μM and [RuRe_2_] ca. 44 μM. In all the experiments carried out, BIH was used as a two-electron donor sacrificial agent. Details on the techniques used to analyze the catalysis products, and other experimental aspects are reported in the Supplementary Information.

#### RuRe2A

**Figure 2:**
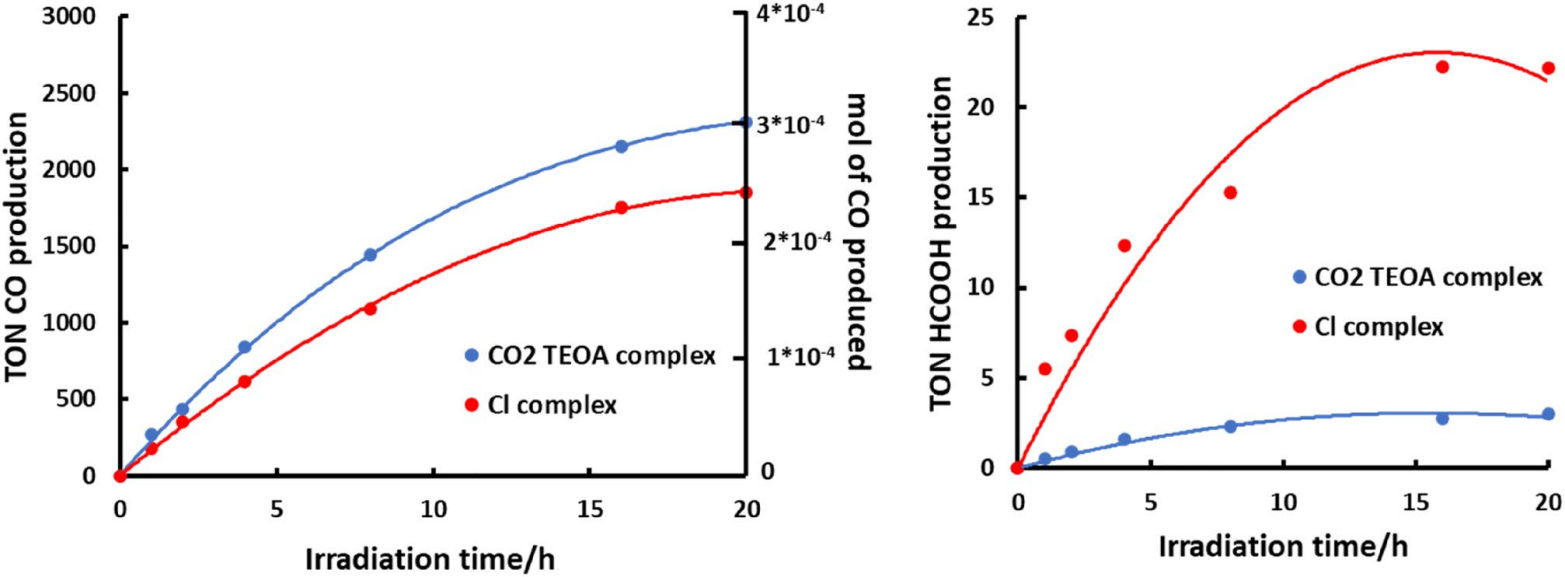
Left: Photocatalytic formation of CO concerning **RuRe2A** (blue line) compared to **RuRe2** (red line) as a function of irradiation time (excitation wavelength, 530 nm). Both complexes are about 50 μM, in a CO_2_-saturated DMA–TEOA (5:1 v/v, 3 mL) solution containing BIH (0.1 M). Right: HCOOH produced.

For the photoreaction studies, solutions (3 mL) of **RuRe2A** (44 μM) and BIH (0.1 M), in DMA-TEOA 5:1 v/v have been charged in several identical tubes in which CO_2_ have been bubbled for 30 min, to obtain saturated CO_2_ solutions. The obtained solutions have been irradiated by using a LED light source (530 nm) at room temperature. The photoreaction was monitored for 45 h. The experiment so prepared allowed to obtain 3 × 10^–4^ mol of CO, corresponding to a turnover number for CO production (TON_CO_) of 2368, based on the amount of **RuRe2A** used (Fig. 2, Table 1). Interestingly, the photoreaction reached a plateau after around 20 h of irradiation time, without further appreciable changes during successive 25 h of irradiation (not shown). Such a behaviour has been attributed to the total consumption of the sacrificial agent within the first 20 h of irradiation. Indeed, from the 1:1 stoichiometric ratio between BIH and CO_2_ for the CO production, it is possible to calculate that after 20 h of irradiation ca. 3 × 10^–4^ mol of CO could be produced, at the best, in the presence of the amount of starting BIH present in solution. This means that the photocatalytic process is essentially quantitative, and the effective limiting agent for CO_2_ photoreduction is the sacrificial agent. Furthermore, the calculated selectivity of CO formation in these conditions was *Γ*_*CO*_ = 99.9%, with no H_2_ and negligible amount of HCOOH observed (see Fig. 2, right panel).

**Table 1:** Photocatalytic properties of **Ru2ReA** and **RuRe2A**. The same properties of **Ru2Re** and **RuRe2** are shown for comparison purposes.

Photocatalyst	Mmol CO produced (percentage of CO production)^a^	TON (CO produced)^b^	Г_CO_
RuRe2A^c^	0.30 (100%)	2368	99.9%
Ru2ReA	0.19 (63%)	2695	98.9%
RuRe2^d^	0.28 (93%)	1850	98.8%
Ru2Re^d^	0.18 (60%)	2486	90%

CO_2_-saturated DMA-TEOA (5:1 v/v), 3 mL solutions containing the supramolecular photocatalysts and a BIH as the sacrificial electron donor (0.1 M). Light irradiation wavelength, 530 nm. The concentration of the photocatalysts containing one Ru-based subunit was 50 µM, whereas the concentration of the photocatalysts containing two Ru-based subunits was 25 µM, unless otherwise stated, to normalize for light absorption in the various experiments. ^a^In parenthesis, the percentage of CO production vs sacrificial reagent concentration is given; based on the concentration of the sacrificial donor, the theoretical maximum of CO photoproduced is 0.30 mmol. ^b^Turnover numbers are based on the amount of photocatalyst. ^c^Concentration of the photocatalyst was 44 µM. ^d^Data from ref.^29^.

#### Ru2ReA

**Figure 3:**
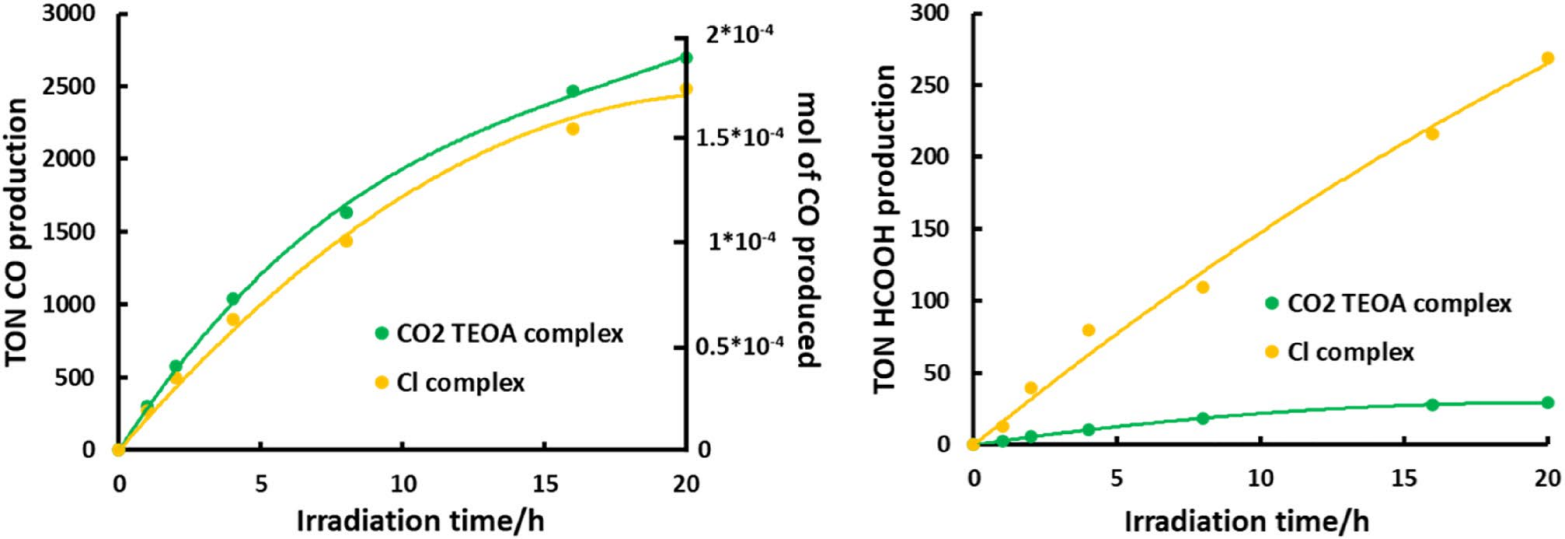
Left: Photocatalytic formation of CO concerning **RuRe2A** (green line) compared to **RuRe2** (orange line) as a function of irradiation time (excitation wavelength, 530 nm). Both complexes are about 25 μM, in a CO2-saturated DMA–TEOA (5:1 v/v, 3 mL) solution containing BIH (0.1 M). Right: HCOOH produced.

A procedure similar to that formerly described for **RuRe2A** was used to study the photocatalytic activity of **Ru2ReA**. 3 mL of a CO_2_-saturated solution of DMA-TEOA 5:1 v/v, containing **Ru2ReA** and BIH with a concentration of 24 μM and 0.1 M respectively—the concentration of **Ru2ReA** is roughly half concentration than that in the photocatalytic system using **RuRe2A** described above, to have the same light absorption, mainly due to the Ru-based subunits—was charged in several identical tubes. The solutions were irradiated by using the same LED light source (530 nm) used in the previous experiment, at room temperature. The photochemical reaction has been monitored for 20 h and a TON_CO_ of 2695 has been calculated within this time, based on the amount of **Ru2ReA** used (Fig. 3, Table 1). The total CO production has been calculated to be 1.9 × 10^–4^ mol, to be compared with 3 × 10^–4^ mol of BIH starting amount. From such data we can state that ca. the 60% of the BIH initially present in solution was consumed after 20 h of irradiation. However, in this case the CO formation continued even after 20 h of irradiation (not shown), suggesting that the photocatalyst was still active. The calculated selectivity of CO formation (*Γ*_*CO*_) in the condition used was ca. 98.9% (Table 1), with small amount of H_2_ and HCOOH present in solution (Fig. 3, right panel).

For both the studied complexes, the results can be considered a significative improvement if compared to the ones obtained in a precedent work (see data in Table 1)^29^ in which both in **RuRe2** and **Ru2Re** species the Re(I) metal ion was coordinated by chloride ion instead of TEOA-CO_2_. Indeed, as far as **RuRe2A** is concern^29^ed, the selectivity and the TON_CO_ values rises by ca. 8 and 21% respectively compared to **RuRe2** complex, whereas for **Ru2ReA** the selectivity and the TON_CO_ values rises by ca. 9 and 8%, respectively, compared to **Ru2Re**. For better comparison, the photocatalytic activity of the parent species **RuRe2** and **Ru2Re**, already reported^24^, are also shown in Figs. 2 and 3, respectively, under the identical experimental conditions.

Comparisons of the photocatalytic data shown in Table 1 and Figs. 2 and 3 suggest some considerations. First, **RuRe2A** and **RuRe2** yield CO almost quantitatively, considering the maximum possible yield of CO on the basis of the sacrificial reagent which is present. Particularly, the CO photoproduction of **RuRe2A** is complete in 20 h, with an extremely high selectivity (Fig. 2). Turnover number values, which are apparently larger for the species containing two photosensitizers and one catalyst units, are less informative on the real efficiency of the compounds in the present cases. In fact, the TONs of **RuRe2A** and **RuRe2** are close to the maximum values possible (in particular for **RuRe2A**, the TON is practically the maximum value reachable under the experimental conditions) already after 20 h of activity; more instructive is the consideration that, although TONs are apparently higher, indeed the percentage of photoconversion of CO_2_, on the basis of the amount of sacrificial reagent needed to sustain catalysis, is around 60% for **Ru2ReA** and **Ru2Re,** whereas it is close to 100% for **RuRe2A** and **RuRe2** (Table 1).

The main difference between the two sets of compounds (that is **Ru2ReA** and **Ru2Re** vs **RuRe2A** and **RuRe2**) is the photosensitizer/catalyst ratio: apparently, to have a single light-harvesting unit (photosensitizer) connected to two catalyst units leads to a more efficient (and selective) photocatalysis. The first events of the photocatalytic process in the studied systems, upon light harvesting, involve the reductive quenching of the triplet metal-to-ligand charge-transfer (MLCT) excited state of the Ru(II) subunit by BIH and successive electron transfer from the reduced Ru-based component to the Re(I) catalytic center(s) [see Eqs. (1)–(3)]. Since **RuRe2A** and **RuRe2** contain two Re(I) centers, the rate constant of electron transfer from the reduced Ru(II) subunit to the Re(I) center(s) is expected to be roughly twice than in **Ru2ReA** and **Ru2Re**, respectively. However, it should be considered that, due to the close reduction potentials of Ru-based and Re-based subunits, the intramolecular Ru-to-Re electron transfer is reversible and equilibration can take place. Actually, the first reduction potential of [Re(dmb)(CO)_3_Cl] (bmp = 4,4′-methyl-2,2′-bipyridine), a model for the Re-based subunit, is − 1.78 V vs Ag/AgNO_3_ in acetonitrile, 10 mV more negative than that of [Ru(dmb)_3_]^2+^ (− 1.77 V), a model for the Ru-based subunit^30^. On the other hand, the first reduction potential of the Re-subunit containing the TEOA-CO_2_ adduct is − 1.74 V, that is 30 mV more positive than that of [Ru(dmb)_3_]^2+^^27^. In all cases, since intramolecular electron transfer is faster than the CO_2_ reduction reaction of the Re catalyst^27^, thermal equilibration between Ru-reduced and Re-reduced subunits in the supramolecular photocatalyst discussed here can occur. Just on simple statistical basis, in **RuRe2A** (as well as in **RuRe2**) the equilibrium in the one-electron reduced form is displaced towards the reduced Re subunits, whereas the contrary occurs for **Ru2ReA** (and **Ru2Re**). For **RuRe2A** and **RuRe2**, this translates into a decreased efficiency of any competitive, poisoning process which could involve the one-electron reduced form of the Ru-based photosensitizer(s), having the final effect of deactivating the photosensitizer itself (in case of irreversible process) towards CO_2_ photocatalysis leading to CO formation. An important competitive process involving the reduced Ru-based photosensitizer is ligand dissociation. Indeed, it is known^31^ that a bpy-type ligand tends to leave the coordination sphere of Ru(II) photosensitizers, with formation of a *solvento* complex, under photocatalytic conditions: such a *solvento* complex (a [Ru(BL)(LL)(L)_2_]^2+^ compound in the present case, where L is a coordinating solvent and where BL and LL are bridging and terminal polypyridine ligands, respectively) behaves as a catalyst for HCOOH formation, and is responsible for the small amount of formate formation, competitive with CO formation, so affecting the selectivity of the photocatalysis^5, 24^. Actually, the amount of HCOOH produced (see Figs. 2 and 3) was clearly higher in the photocatalytic reactions with **Ru2ReA** (and **Ru2Re**) than with **RuRe2A** (and **RuRe2**). With the assumption that the reduced form of the Ru-based PS is the main precursor of the *solvento* complex—corroborated by the lower threshold for bpy photodissociation in the singly reduced Ru(bpy)_3_^+^ with respect to Ru(bpy)_3_^2+^, although these data refer to the gas phase^32, 33^—reduced probability to localize the unpaired electron of the one-electron reduced form of the supramolecular photocatalysts on the Ru unit would be at the root for improved yield and selectivity of photocatalytic CO formation for **RuRe2A** and **RuRe2** compared to the other two species. In fact, although photodissociation of a bpy ligand in Ru-based subunit is also possible in coordinating solvents^14–16^, this process would be much more favored for the reduced form of the Ru-based photosensitizer^32, 33^. These results also indirectly confirm that the rate-determining step in this CO_2_ photoreduction experiments is the catalytic cycle involving the Re subunit(s), as previously reported^27^.

**Figure 4:**
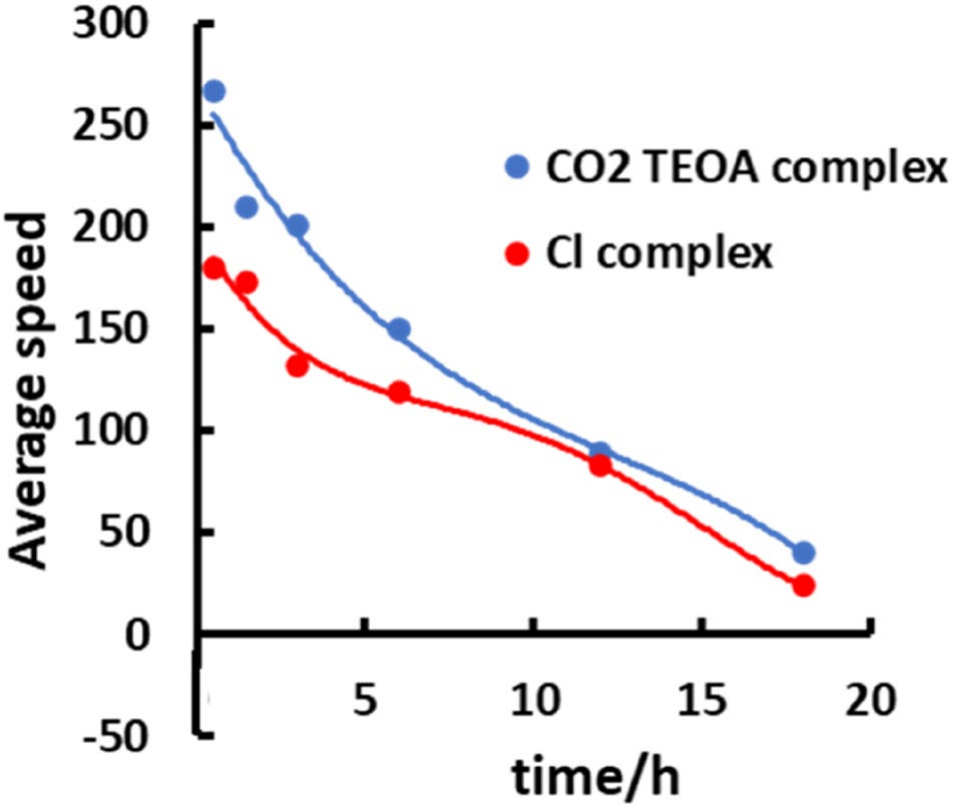
Average speed of CO formation for **RuRe2A** (blue line) and the parent reference compound **RuRe2** (red line), as a function of time. The averaged rates at various times are calculated by averaging CO production at various intervals of time, based on the data in Fig. 2 (see further details in the main text). The shown curves are guides for the eyes, not best fitting.

As far as the difference in the photocatalytic behavior of **RuRe2A** and **Ru2ReA** vs **RuRe2** and **Ru2Re** is concerned, comparison between the photocatalytic formation of CO curves of the new species and their respective parent compounds in Figs. 2 and 3 indicates that the compounds **RuRe2A** and **Ru2ReA**, containing preformed adducts, are more efficient photocatalytic systems, particularly at the beginning of the photocatalysis. This is exemplified by Fig. 4, which shows the rates of CO photoproduction for **RuRe2A** and **RuRe2** at different times during the process.

In this figure, the apparent rates at different times are calculated by averaging CO production at various intervals of time: for example, the apparent rate of **RuRe2A** at 3 h is calculated from the amount of CO formation in the interval 2–4 h of photoproduction, from the data shown in Fig. 2. From Fig. 4, it appears that there is a significative difference in the rate of CO formation for **RuRe2A** and **RuRe2**, particularly during the initial stage of the process. With CO formation delayed for Cl-containing supramolecular photocatalysts, stability of the photosensitizers units is also comparatively decreased, and a larger amount of Ru-based *solvento* complex is expected to be formed, so affecting overall photocatalysis selectivity and yield. Although smaller with time, the difference in apparent rate of CO formation is still present between **RuRe2A** and **RuRe2** during all the photocatalytic processes investigated. The difference is once more attributed to the different percentage of Ru-based vs Re-based electron localization in the one-electron reduced state of the species: as mentioned above, the reduction potential of the Re-based subunit(s) containing CO_2_-TEOA adduct (that is, in **RuRe2A**) is 30 mV less negative than that of the Ru-based subunit, whereas the reduction potential of the Re-based subunit(s) of **RuRe2** is almost identical (or even more negative, see above) to that of the Ru-based subunit. This would result in the one-electron reduced species of the Cl-containing complex having a greater tendency for unpaired electron localization on the Ru subunit compared to the one-electron reduced species of the CO_2_-TEOA adduct-containing compound, so making **RuRe2** easier to decompose forming the *solvento* complex, with decreased CO production rate. The presence of chloride anions in solution, which could interfere with adduct formation when CO_2_ is reduced to CO and new adduct must be continuously formed, could also contribute to slow down CO formation for **RuRe2** in comparison to **RuRe2A** during the photocatalysis. The different rates of CO formation at initial stages of the photocatalytic process, balanced by a larger amount of formate production for **RuRe2** (see Figs. 2 and 3), as well as our interpretation, would suggest that in **RuRe2** (and in **Ru2Re**) the starting material still contains, at least in part, chloride anion as ligand, that is an equilibration between CO_2_-TEOA adduct-containing and Cl-containing species occurs, in spite of the large excess of TEOA in solution compared to chloride. An induction period for photocatalysis, due to CO_2_TEOA insertion, is not observed for **RuRe2** or **Ru2Re**, but the induction period could be masked by the photoreaction involving the species containing the formed adduct. Since the chloride species would be more prone to bpy dissociation on the Ru-based subunit according to the above discussion, the overall CO formation would be lower during all the photocatalytic process, in parallel with a larger amount of formate production (the increased amount of *solvent*o complex formed at the initial stage would affect all the course of the photocatalysis).

## Conclusions

Two new multicomponent, supramolecular photocatalysts containing Ru(II) polypyridine units as light-harvesting photosensitizers and Re(I) polypyridine subunits as catalytic centers have been prepared. The species, named **RuRe2A** and **Ru2ReA**, differs from recently investigated parent **RuRe2** and **Ru2Re** compounds because the coordination sphere of the catalytic Re(I) subunits contain the preformed CO_2_TEOA adduct (known to be the effective catalytic subunits) instead of chloride ligands. Comparison between the photocatalytic properties of the new compounds and those of their previously studied parent compounds allows to investigate the effect of the CO_2_TEOA insertion process as well as the eventual effect of the presence of chloride ions in solution (inevitable for **RuRe2** and **Ru2Re**) on the photocatalytic processes, an issue which has been unexplored and has limited direct comparison of supramolecular catalysts reported in literature.

**RuRe2A** and **Ru2ReA** overcome the photocatalytic properties of the parent compounds, exhibiting quite efficient and selective photoreduction of CO_2_ to CO, with outstanding TONs of 2368 and 2695 and a selectivity of 99.9% and 98.9%, respectively, on an irradiation time of 20 h. Quite interestingly, for **RuRe2A** photoreduction of CO_2_ to CO occurs quantitatively, by considering the amount of sacrificial donor which is present in solution. The results also confirmed that better results are obtained for a 1:2 photosensitizer-catalyst ratio in the supramolecular photocatalyst structure, analogously to what found for formerly reported parent species^29^. This effect is attributed to the distribution of the one-electron reduced form of the photocatalysts, which favours localization on the Ru-based subunits in the **Ru2ReA** (and **Ru2Re**), with concomitant increase of the probability of ligand loss on the photosensitizer so leading to increasing competitive formate production over CO production.

The improved photocatalytic properties of the new compounds **RuRe2A** and **Ru2ReA** compared with those of the parent **RuRe2** and **Ru2Re** species, particularly during the initial stage of the photocatalysis process, are attributed to the unavoidable presence of chloride ions in solution for **RuRe2** and **Ru2Re**, which could interfere with the CO_2_TEOA adduct formation, a needed requisite for CO forming catalysis. These results strongly indicate the utility of preparing supramolecular photocatalysts containing preformed adducts.

## Supplementary Information

Supplementary Information.

## Data Availability

All data generated or analysed during this study are included in this published article and in its Supplementary Information.
