# Supplementary material for: Photocatalyzed CO2 reduction to CO by supramolecular photocatalysts made of Ru(II) photosensitizers and Re(I) catalytic subunits containing preformed CO2TEOA adducts
Source: Sci Rep. 2023 Jul 13;13:11320. doi: 10.1038/s41598-023-38411-3 (PMC10344947; doi:10.1038/s41598-023-38411-3)
Supplement: Supplementary file 1 — Supplementary Information. [file 41598_2023_38411_MOESM1_ESM.docx]

**Photocatalyzed CO_2_ reduction to CO by supramolecular photocatalysts made of Ru(II) photosensitizers and Re(I) catalytic subunits containing preformed CO_2_TEOA adducts**

Antonio Santoro, Ambra M. Cancelliere, Kei Kamogawa, Scolastica Serroni, Fausto Puntoriero, Yusuke Tamaki, Sebastiano Campagna, Osamu Ishitani

**Supplementary Information**

**General procedures and materials**

DMA was dried on 4 Å molecular sieves at room temperature and distilled at reduced pressure before use. The TEOA was distilled under reduced pressure (<133 Pa). The distilled solvents were stored under an Ar atmosphere. All other reagents were of reagent grade and used without further purification. The UV-Vis absorption spectra were measured using a JASCO V-670 spectrophotometer. FT-IR spectra were recorded on a JASCO FT-IR 6600 spectrophotometer.

Electrospray ionization time-of-flight mass spectroscopy (ESI-TOFMS) was undertaken with a Waters LCT Premier, with acetonitrile as mobile phase.

**Photocatalytic reactions**

TON determinations were carried out by using DMA–TEOA (5:1 v/v, 3 mL) solutions containing the photocatalyst and BIH (0.1 M) in Pyrex test tubes (11 mL volume) that were purged with CO_2_ for 30 min and subsequently irradiated at 490–620 nm (λmax = 530 nm) using the Iris-MG merry-go-round irradiation apparatus with an LED light source (CELL System Co.).

All the gaseous photoreaction products were quantified taking advantage of a GC-TCD (GL science GC323) equipped with a column of active carbon and argon as gas carrier. The amount of formic acid was quantified by a capillary electrophoresis system (Agilent 7100L) with buffer solution and water dilution.

**Absorption spectra**

Figures S1 and S2 show the absorption spectra of **RuRe2A** and **Ru2ReA** in a CO_2_-saturated DMA–TEOA (5:1 v/v, 3 mL) solution containing BIH (0.1M), before irradiation and during photocatalysis. The initial absorption spectrum of each compound is dominated in the visible by the spin-allowed metal-to-ligand charge-transfer (MLCT) transition involving the Ru(II) subunits, according to the usual properties of Ru(II) polypyridine complexes [S1, S2, S3], which obscure the much less intense MLCT band involving the Re(I) subunits, expected to be in the same spectral region [S4, S5]. The spectral changes upon irradiation are consistent with bipyridine (bpy) photorelease from the Ru(II) centers, with formation of the solvato complex. In fact, bpy substitution with DMA leads to MLCT shift to lower energy [S1], with increased absorption in the 500-600 nm region and decreased absorption of the initial band at 440-460 nm.


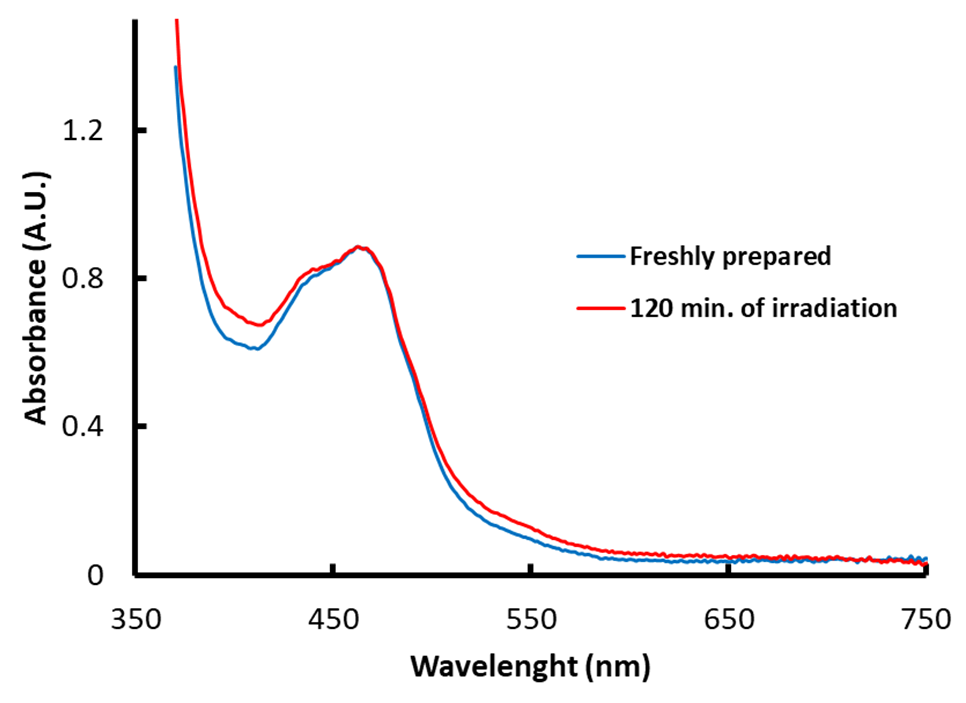


Figure S1. Absorption spectra of **RuRe2A** in CO_2_-saturated DMA–TEOA (5:1 v/v, 3 mL) solution containing BIH (0.1M), before and during photocatalysis.


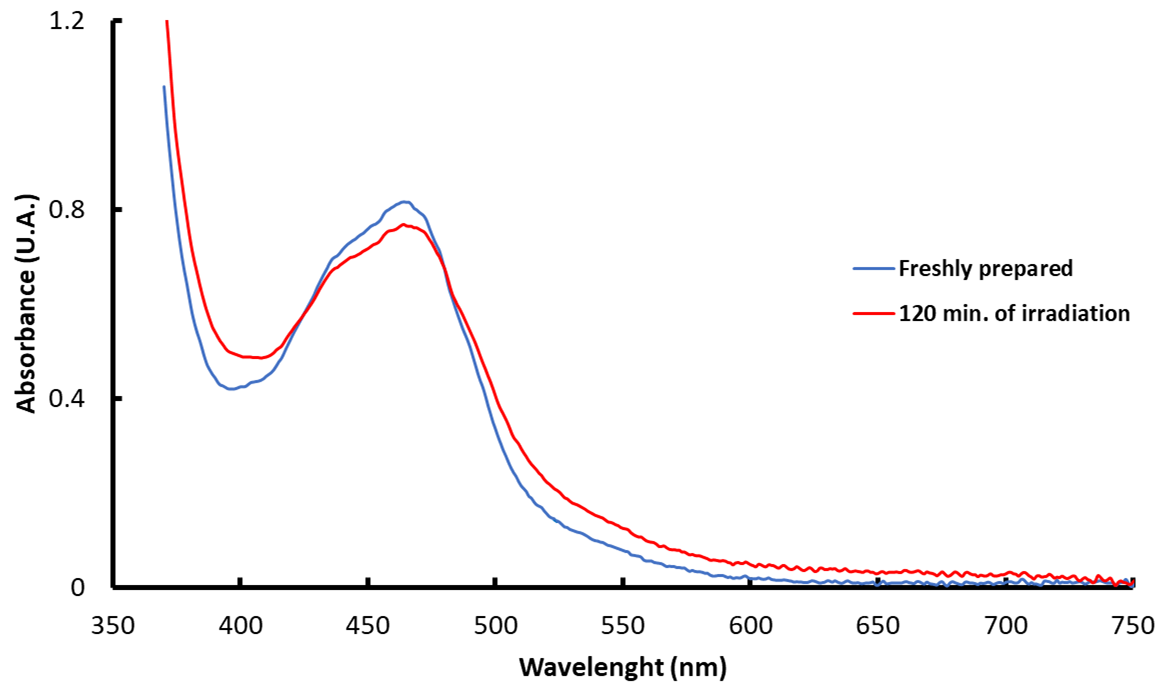


Figure S2. Absorption spectra of **Ru2ReA** in CO_2_-saturated DMA–TEOA (5:1 v/v, 3 mL) solution containing BIH (0.1M), before and during photocatalysis.

**Synthesis**

The ligand and the precursor complexes **Ru**, **Ru2**, **Ru2Re** and **RuRe2** were reprepared by using a procedure already reported by the authors [S6]. The structural formula of **Ru** and **Ru2** are shown in Figure S3. **Ru2Re** and **RuRe2** structures are shown in the main text.

Here we report the synthesis and characterization data for the studied **Ru2Re** and **RuRe2** species. The protocol procedures for the preparation of **Ru2ReA** and **RuRe2A** are reported in the main text.

Figure S3. *Structural formulas of the* ***Ru*** *and* ***Ru2*** *species. All the metal complexes are hexafluorophosphate salts.*

**Synthesis of Ru2Re**

**Ru2** (50.6 mg, 0.023 mmol) and Re(CO)5Cl (8.9 mg, 0.025 mmol) were dissolved in 30 mL of 1,2- dichloroethane for 6 h at reflux under argon atmosphere. After this period, the product was precipitated by addition of NH4PF6 and filtered out. The isolated orange solid was washed several times with diethyl ether. Yield: 60%. ESI-TOFMS (in MeCN) m/z: Calculated for C96N14H86ClO3Ru2Re (M – 4 PF6) 478.1170, found 478.1147. FT-IR (in CH3CN) νCO∕cm−1: 2020, 1915, 1895.

**Synthesis of RuRe2**

**Ru** (20 mg, 0.017 mmol) and Re(CO)5Cl (12.7 mg, 0.035 mmol) were dissolved in 32 mL of 1,2- dichloroethane for 4 h at reflux under argon atmosphere. After this period, the product was precipitated by addition of NH4PF6 and filtered out. The isolated orange solid was washed several time with diethyl ether. Yield: 92%. ESI-TOFMS (in MeCN) m/z: Calculated for C75N10H64Cl2O6RuRe2 (M – 2 PF6) 874.1349, found 874.1369. FT-IR (in CH3CN) νCO∕cm−1: 2021, 1915, 1896.

**References to Supplementary Information**

[S1] Juris, A.; Balzani, V.; Barigelletti, F.; Campagna, S.; Belser, P. & Von Zelewsky, A. Ruthenium(II) Polypiridine Complexes: Photophysics, Photochemistry, Electrochemistry, and Chemiluminescence. *Coord. Chem. Rev.*, **1988**, *84*, 85-277.

[S2] Meyer, T. J. Photochemistry of metal coordination complexes: metal to ligand charge transfer excited states. *Pure Appl. Chem.*, **1986**, *58*, 1193-1206.

[S3] Campagna, S.; Puntoriero, F.; Nastasi, F.; Bergamini, G. & Balzani, V. Photochemistry and photophysics of coordination compounds: ruthenium. *Top. Curr. Chem.*, **2007**, *280*, 117-214.

[S4] Juris, A.; Campagna, S.; Bidd, I.; Lehn, J.-M. & Ziessel, R. Synthesis and photophysical and electrochemical properties of new halotricarbonyl(polypyridine)rhenium(I) complexes. *Inorg. Chem.*, **1988**, *27*, 4007.

[S5] Kirgan, R. A.; Sullivan, B. P. & Rillema, D. P. Photochemistry and photophysics of coordination compounds: rhenium. *Top. Curr. Chem.*, **2007**, *281*, 45-100.

[S6] Cancelliere, A. M.; Puntoriero, F.; Serroni, S.; Campagna, S.; Tamaki, Y.; Saito, D. & Ishitani, O. Efficient trinuclear Ru(II)–Re(I) supramolecular photocatalysts for CO2 reduction based on a new tris-chelating bridging ligand built around a central aromatic ring. *Chem. Sci.* **2020,** *11*, 1556-1563.
